# High saturated-fat and low-fibre intake: a comparative analysis of nutrient intake in individuals with and without type 2 diabetes

**DOI:** 10.1038/nutd.2014.2

**Published:** 2014-02-03

**Authors:** C Breen, M Ryan, B McNulty, M J Gibney, R Canavan, D O'Shea

**Affiliations:** 1 Diabetes and Endocrine Units, St Columcille's and St Vincent's University Hospitals, Dublin, UK; 2 Institute of Food and Health, University College Dublin, Dublin, UK

**Keywords:** type 2 diabetes, nutrient intake, food intake, recommendations, education

## Abstract

**Objective:**

The aim of dietary modification, as a cornerstone of type 2 diabetes (T2DM) management, is to optimise metabolic control and overall health. This study describes food and nutrient intake in a sample of adults with T2DM, and compares this to recommendations, and to intake in age, sex, body mass index (BMI) and social-class matched adults without T2DM.

**Design:**

A cross-sectional analysis of food and nutrient intake in 124 T2DM individuals (64% male; age 57.4±5.6 years, BMI 32.5±5.8 kg m^−2^) and 124 adults (age 57.4±7.0 years, BMI 31.2±5.0 kg m^−2^) with no diabetes (ND) was undertaken using a 4-day semiweighed food diary. Biochemical and anthropometric variables were also measured.

**Results:**

While reported energy intake was similar in T2DM vs ND (1954 vs 2004 kcal per day, *P*=0.99), T2DM subjects consumed more total-fat (38.8% vs 35%, *P*⩽0.001), monounsaturated-fat (13.3% vs 12.2% *P*=0.004), polyunsaturated-fat (6.7% vs 5.9% *P*<0.001) and protein (18.6% vs 17.5%, *P*⩽0.01). Both groups exceeded saturated-fat recommendations (14.0% vs 13.8%). T2DM intakes of carbohydrate (39.5% vs 42.9%), non-milk sugar (10.4% vs 15.0%) and fibre (14.4 vs 18.9 g) were significantly lower (*P*<0.001). Dietary glycaemic load (GL) was also lower in T2DM (120.8 vs 129.2; *P*=0.02), despite a similar glycaemic index (59.7 vs 60.1; *P*=0.48). T2DM individuals reported consuming significantly more wholemeal/brown/wholegrain breads, eggs, oils, vegetables, meat/meat products, savoury snacks and soups/sauces and less white breads, breakfast cereals, cakes/buns, full-fat dairy, chocolate, fruit juices, oily fish and alcohol than ND controls.

**Conclusion:**

Adults with T2DM made different food choices to ND adults. This resulted in a high saturated-fat diet, with a higher total-fat, monounsaturated-fat, polyunsaturated-fat and protein content and a lower GL, carbohydrate, fibre and non-milk sugar content. Dietary education should emphasise and reinforce the importance of higher fibre, fruit, vegetable and wholegrain intake and the substitution of monounsaturated for saturated-fat sources, in energy balanced conditions.

## Introduction

Type 2 diabetes (T2DM) has reached epidemic proportions worldwide, affecting an estimated 55.2 million adults in Europe alone.^1^ It carries with it an increased mortality risk, multiple comorbidities, decreased quality of life and a significant economic burden. Diet remains the cornerstone of effective T2DM management and encouraging the adoption of a lifelong healthy diet, which optimises metabolic control as the ultimate aim of dietary interventions.^2^ Maintaining energy balance is one of the most important and effective therapeutic challenges in overweight/obese individuals. When a negative energy balance is achieved, glycaemic control, lipid levels, blood pressure and mortality risk all improve.^3^

The optimal dietary macronutrient composition for achieving energy balance in T2DM remains controversial. Studies investigating the relationship between macronutrients and metabolic control are contradictory, leading recent Diabetes UK (DUK) guidelines^4^ to conclude that there is currently little evidence to support any one ‘ideal' macronutrient composition diet in T2DM. Furthermore, the involvement of individual food groups in the inherent relationship is unclear.^5^ The Diabetes and Nutrition Study Group (DNSG) of the European Association for the Study of Diabetes (EASD)^6^ recommend intakes that are similar to the World Health Organisation dietary reference intakes for the general population,^7^ that is, low total (<35%) and saturated- (<10%) fat, moderate protein (10–20%) and moderate-to-high carbohydrate (45–60%) intakes. Clinical guidelines also emphasise overall dietary quality: encouraging a wide variety of nutrient-dense low glycaemic index (GI) foods including fruit, vegetables, legumes and wholegrain cereals, a moderate free sugar intake (up to 10% of energy) and 10–20% monounsaturated-fat (MUFA).^6^

Despite evidence demonstrating the benefits of dietary modification on metabolic control in T2DM, the proportion of adults achieving the desired dietary targets is known to vary considerably.^8^ Detailed descriptions of food group selection and their impact on nutrient intake and achievement of dietary targets in T2DM, however, has not been widely evaluated. Given that dietary advice must be food based, it is essential that diabetes educators have a comprehensive understanding of food group selection in T2DM, what impact these choices have on nutrient intakes and how these choices may differ to those of individuals without diabetes. The aim of the current study is therefore to provide a detailed description of food group selection and nutrient intake in a sample of adults with T2DM, and to compare this to intake in age, sex, body mass index (BMI) and social-class matched adults without T2DM. A comparison is also made with current dietary guidelines.

## Materials and methods

### Participants

One hundred and twenty-four adults with T2DM were recruited from the Diabetes Service, St Columcille's Hospital, Dublin, Ireland between January 2011 and June 2012. Eligible candidates were identified and contacted via letter and telephone by the researcher (CB). In addition, advertising posters for the study were displayed in waiting areas in the Diabetes Clinic. Two hundred and forty-three patients were approached/screened, with 124 meeting the eligibility criteria and consenting to take part. Sample size is similar to other studies in the area.^9^ Participants were over 18 years of age, were diagnosed with T2DM at least 6 months previously and were not pregnant/lactating. Ethical approval for the study was granted by the Ethics and Medical Research Committee, St Vincent's Healthcare Group. All patients gave written informed consent before participation.

Upon entering the service, all T2DM subjects had received standard dietary advice for T2DM, to follow a healthy eating plan. In Ireland, healthy eating advice in T2DM is based on the Food Pyramid.^10^ This tool promotes a wide variety of portion controlled foods including fruit and vegetables, wholegrain cereals, low-fat dairy and protein foods, in the context of a 45–60% carbohydrate, <35% fat and <10% saturated-fat diet. High fat, sugar, salt foods such as confectionary are recommended only in moderation.^10^ One hundred and eighteen individuals (95%) recalled and self-reported the setting in which this education was delivered: 53% in a group education setting delivered jointly by a dietitian and diabetes nurse specialist (DNS) and 42% in a one-to-one setting with dietitian and/or DNS. Sixty-three per cent of participants self-reported that it was longer than 12 months as they last received dietary advice regarding T2DM from a health-care professional (HCP).

A control group of 124 adults with no diabetes (ND) matched for age, sex, BMI and social class were selected from the database of the Irish National Adult Nutrition Survey (NANS). The NANS investigated habitual food and beverage consumption, lifestyle and health indicators in a nationally representative sample of 1500 adults in the Republic of Ireland between 2008 and 2010, as described previously.^11^ Participants were free-living adults who were not pregnant/lactating. The survey was approved by the Clinical Research Ethics Committee of the Cork Teaching Hospitals, University College Cork and the Human Ethics Research Committee of University College Dublin and conducted according to the guidelines laid down in the Declaration of Helsinki.^11^

### Dietary assessment

Procedures for the measurement, assessment and analysis of food intake in the T2DM subjects were identical to those used in the NANS.^11^ Participants were asked not to alter their usual dietary intake and to record all food and beverages consumed over a consecutive 4-day period, which included at least 1 weekend day. Participants were asked to give as much details as possible regarding the types and brands of foods in addition to cooking and preparation methods. Data on the timing and location of each eating occasion were also recorded, along with noting of any significant leftovers. Participants quantified food intakes using a portable food weighing scales (Tanita KD-400; Tanita, Tokyo, Japan), using the manufacturer's information on food packaging and/or using household measures (cups, tablespoons, etc). In addition participants were asked to retain the outer wrappers of packaged foods, in order to later provide comprehensive information on food composition and portion size. Before commencing the assessment, the researcher met with participants and gave detailed instructions for completion of the diary and use of the scales. Each participant was contacted on day 2 of the food diary to review progress, check for completeness and clarify any missing details regarding food descriptors or quantities. When the diary was completed, the researcher again met with each participant and further clarified details using a photographic food atlas. The majority of food was consumed inside the home for both the T2DM (85.4%) and the ND group (84.9%). Food intake was quantified from weights or manufacturers information (59.4% vs 61.9%), using a food atlas (14.6% vs 12.8%), from average portion sizes (13.7% vs 15.2%), household measures (8.3% vs 9.4%) or estimated (3.2% vs 1.5%) for T2DM vs ND groups, respectively.

Dietary data was analysed using Weighed Intake Software Programme (WISP) (Version 3; Tinuviel Software, Llanfechell, UK), which contains food composition data derived from the 5th and 6th edition of McCance and Widdowson's Food Composition Tables plus all nine supplemental volumes.^12, 13^ In addition, modifications were made to the food composition database to include composite dishes, nutritional supplements and generic Irish foods.^14^ In cases where the portion size was not detailed sufficiently, average food portion sizes^11, 15^ were used or quantities estimated by the researcher based on their knowledge of the respondent's general eating habits as observed during the recording period, as detailed elsewhere.^11^ The research dietitian was solely responsible for the quantification, coding, and entry and checking of data for T2DM subjects, using an identical guideline to that used by NANS researchers. A food intake database was extracted from WISP, comprising over 21 900 rows of data that contained the nutrient breakdown for each item consumed, by each of the 248 participants (cases and controls), at each eating occasion, for each of the four recording days. All foods consumed were aggregated into 38 mutually exclusive food groups (Supplementary Appendix 1). These food groups were similar to those used in the Irish NANS^11^ and by McGowan and McAuliffe^16^ when examining food intakes in pregnant Irish women, with food groups aggregated or divided as appropriate to enhance analysis of the data in line with diabetes dietary guidelines (e.g. a ‘breakfast cereals' group was broken down to ‘refined breakfast cereals' and ‘wholegrain breakfast cereals' groups).

The food intake database contained GI values of foods, each of which was manually checked against the most up-to-date published GI values available.^17^ Where more than one GI value was found matching the foods description, a mean value was used. Where available, mean GI values from studies carried out in the United Kingdom were used as these values were considered to be more representative of foods commonly consumed in Ireland. For foods without a GI value, the GI value of a closely equivalent food with a similar nutritional composition was imputed. Foods with a carbohydrate content of ⩽5 g per 100 g were given a GI value of zero. For composite meals/recipes, the GI value of the predominant carbohydrate source or the mean GI value of multiple carbohydrate sources was used. If no GI value could be assigned using the above methodology, a GI value of 50 was assigned.^18^ Dietary GI was calculated as the sum of the weighted GI, with the weighting proportional to the contribution of the food to total carbohydrate intake (GI of the food × the carbohydrate content of the food divided by the total carbohydrate intake/day for each individual). Dietary glycaemic load (GL) was calculated as the sum of the GI of each food times the amount of available carbohydrate (g) per serving.

Resting metabolic rate was estimated using the predictive equation of Henry.^19^ An assessment of energy intake (EI) was conducted using the method of Goldberg *et al.*^20^ showing the ratio of EI to basal metabolic rate (BMR). A ratio of 1.1 was used as the threshold for indicating possible under-reporting in this analysis, as used in previous studies in T2DM.^21^ When compared, neither the mean EI/BMR (1.15±0.32 vs 1.20±0.30, *P*=0.12) or the proportion of subjects reporting an EI/BMR <1.1 (36.3 vs 38.8%, *P*=0.68) differed significantly between T2DM and ND subjects, therefore potential under-reporters were not excluded from the main analysis. Individuals with an EI/BMR ⩽1.1 did not differ significantly from normal energy reporters by age, gender or social class, but were more obese (BMI 31.8 vs 30.5 kg m^−2^, *P*=0.03).

### Anthropometry and lifestyle assessment

All participants completed a Health and Lifestyle Questionnaire,^11^ which collected information on sociodemographics, education levels and smoking status. Data on medication use and previous diabetes education were also collected in the T2DM subjects. Weight was measured to the nearest 0.1 kg, in light clothing and without shoes using a Seca 665 (Seca Ltd, Birmingham, UK) or Tanita BC-420MA (Tanita Ltd, Middlesex, UK). Height was measured to the nearest 0.1 cm using a Seca 242 stadiometer (Seca Ltd, Hamburg, Germany) or Leicester portable height measure (Chasmores Ltd, London, UK). BMI was calculated using the standard formula (weight (kg) height (m^−2^)). Waist circumference was measured in duplicate, at the end of a normal expiration, to the nearest 0.1 cm, at the midpoint between the lowest rib and the iliac crest. Blood pressure was measured in a seated position, at rest, using an automated sphygmomanometer (Omron M6/Hem 6111; Omron Healthcare, Milton Keynes, UK).

### Biochemistry

Blood samples were drawn following an overnight fast. Serum glucose was measured using hexokinase methodology in an automated analyser (Olympus AU640; Olympus, Germany/Rx Daytona, Randox Laboratories, Wülfrath, Germany). Serum total, low-density lipoprotein- and high-density lipoprotein-cholesterol and trigaclycerol concentrations were collected in serum tubes and measured using enzymatic reagents (Olympus AU640; Olympus, Germany/Rx Daytona, Randox Laboratories). In T2DM subjects, serum insulin was measured using an automated monoclonal antibody-based two-site immunoenzymometric assay (AIA-1800 system; Tosoh Europe NV, Tessenderlo, Belgium), and glycosylated haemoglobin was measured with an automated HPLC instrument-reagent system (model HLC-723 G7; Tosoh Europe NV).

### Statistical analysis

Statistical analysis was performed using PASW Statistics Version 18.0 (SPSS Inc., Chicago, IL, USA). Variables were assessed for normality using the Kolmogorov–Smirnov test. Continuous data with a normal distribution is presented as mean±s.d. and differences assessed using a paired *t*-test. Continuous data that was not normally distributed (including all food group intakes) is presented as median and interquartile range, with differences assessed using the Wilcoxon signed-rank test. Percentage differences in categorical variables were assessed using Pearson's *χ*^2^-test. The percentage of participants meeting dietary recommendations was based on recommendations from the DNSG of the EASD,^6^ DUK^4, 6^ and Irish Recommended Daily Allowances.^22^ P⩽0.05 was considered statistically significant.

## Results

### Participant characteristics

The sociodemographic and clinical characteristics of both groups are shown in Table 1. T2DM subjects had significantly higher waist circumference, fasting glucose and trigaclycerol levels and lower total, low-density lipoprotein- and high-density lipoprotein-cholesterol compared with ND individuals.

### Nutrient intake

T2DM subjects reported consuming significantly more total-fat, polyunsaturated-fat (PUFA), MUFA and protein and significantly less carbohydrate, as a percentage of total energy than ND subjects (Table 2). Both groups reported consuming a saturated-fat intake exceeding the current recommendation of <10% of EI (14.0% vs 13.8% for T2DM and ND, respectively). Overall dietary fibre intake was lower in the T2DM group (with only 12.1% of individuals meeting the dietary recommendation of 24 g per day) as was sugar and non-milk sugar intakes (Table 2). Although the overall GI of the diet did not differ between T2DM and ND, the T2DM subjects reported a lower GL.

T2DM subjects reported a significantly higher sodium intake, lower calcium intake and a lower per cent contribution of alcohol to EI. Although there was no absolute difference in vitamin D intake, only 2.5% of T2DM subjects vs 12.9% of ND met the dietary recommendations for vitamin D (Table 2). Vitamin D was the only micronutrient for which the contribution of nutritional supplements to intake differed significantly between the cohorts (4.0±15.1% vs 9.6±22.7%, *P*=0.05, for T2DM and ND, respectively).

Subgroup analysis, excluding individuals with an EI/BMR ⩽1.1, did not alter the significance of the above differences in nutrient intake across the two groups.

### Food group contribution to nutrient intake

As detailed in Table 3, T2DM subjects reported significantly higher intakes of wholemeal/brown and wholegrain breads, eggs, oils, vegetables, meat/meat products, savoury snacks and soups/sauces, while ND subjects reported significantly higher intakes of white breads, breakfast cereals, cakes and buns, full-fat dairy products, chocolate confectionary, fruit juices, oily fish and alcohol.

Staple foods were significant contributors to EI in both groups. Within these there were subtle differences in food group contribution, with wholemeal/brown breads, non-red meats/meat products, potatoes and butter/full-fat spreads all contributing more significantly to EI in the T2DM group (Table 4).

There were differences in the main contributors to carbohydrate intake between the two groups. Wholemeal/brown bread and low-fat milk/yoghurts were significantly greater contributors to carbohydrate intake in T2DM, while the opposite was true for white bread and full-fat milk/yogurts. Fruit, potatoes and wholegrain breakfast cereals contributed significantly to carbohydrate intake in both groups. Cakes/buns and sugars/syrups were significant contributors among the ND subjects, but minimal contributors to carbohydrate intake in T2DM subjects (Table 4).

Non-red meat/meat products and red meat contributed more significantly to overall fat intake in T2DM than in the matched ND controls, as did soups/sauces and wholemeal/brown bread. Butter/full-fat spreads, poultry, eggs and cheeses were significant contributors to fat intake in both groups. Cakes/buns and full-fat milk/yogurts contributed to fat intake in ND subjects, but contributed only minimally to fat intake among T2DM subjects (Table 4).

Food group contribution to protein intake was similar across both groups, with meats, poultry, breads, wholegrain breakfast cereals, eggs and cheeses all among the top 10 contributors to intake in both groups (Table 4).

**Figure 1:**
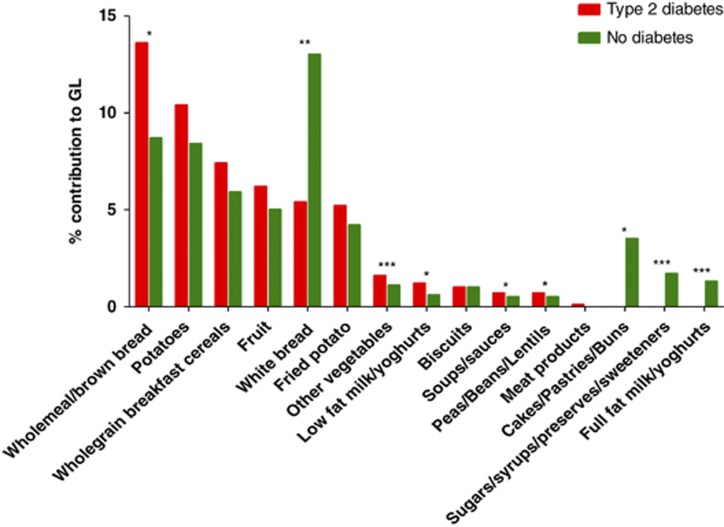
Food group contribution to GL in individuals with T2DM and ND. *P<0.05, **P<0.01, **P<0.001.

Staple carbohydrate foods including breads, potatoes, breakfast cereals, fruit and milk/yogurts contributed significantly to overall GL in both groups. Within the T2DM group, wholemeal/brown bread, vegetables, legumes and low-fat milk/yogurt contributed significantly more, while white bread, cakes/buns, full-fat milk/yogurt and sugars/syrups contributed significantly less to GL than in the ND controls (Figure 1).

## Discussion

This study evaluated food and nutrient intake in a sample of adults with T2DM, and found that this group made different food choices to adults without diabetes. These choices resulted in a high saturated-fat diet, with a higher total-fat, MUFA, PUFA and protein content and a lower GL, carbohydrate, fibre and non-milk sugar content. Although the cross-sectional design of the study limits any conclusions about changes in dietary intake over time as a result of diabetes diagnosis, the study uses high-quality data to give a detailed picture of how food and nutrient intake differs in individuals with T2DM. This information offers potential insights into foods to target during dietary education and the most effective strategies to improve overall dietary quality in T2DM.

In T2DM, total EI remains one of the most important factors to consider for both glycaemic and weight control,^4^ as increased energy consumption directly induces insulin resistance.^23^ Moderate energy restriction results in clinically significant weight loss with concurrent reductions in waist circumference^24^ and has been shown to improve glycaemic control, insulin sensitivity and reduce the need for diabetes medications.^2^ Reported EI did not differ significantly between the case and control subjects in the current study and the removal of energy under-reporters did not alter the results. Increased visceral adiposity, as evidenced by waist circumference (108.0 vs 102.0 cm, *P*<0.001 in T2DM vs ND controls), highlights the necessity for clear messages regarding energy balance, body weight and adipose tissue distribution in T2DM dietary education.

There is much controversy and contradiction in the literature regarding the optimum dietary macronutrient composition for delivering key nutrients under energy balanced conditions in T2DM. This has led to greater focus on concepts of overall dietary quality and broader dietary patterns that promote metabolic health. The Mediterranean dietary pattern, in particular, has been shown to improve glycaemic control in T2DM^25, 26^ and to be effective in achieving weight loss among individuals with T2DM.^26^ This approach is characterised predominantly by foods of plant origin including fruit, vegetables, legumes, nuts, wholegrain cereals and olive oil, with low-moderate dairy, fish and chicken and low consumption of meat/meat products.^27^ Typically fat intake ranges from 25 to 35% of energy, with a proportionately low saturated-fat (<8%) and high MUFA content.^27^ This dietary approach is at odds with the high saturated-fat, low-fibre, western diet that predominated in the current study. A minority of both cohorts (5.7% of T2DM cases and 9.6% of controls) were meeting the <10% energy target for saturated fat. This may be indicative of the wider problem of saturated-fat overabundance in western-diet food supply chains and the need for public health policy change to support healthier choices at a population level.^28^ Reducing saturated fat (butters and meat products in particular were significant contributors in both the T2DM and ND groups) and replacing this with MUFA (monounsaturated oils/spreads and oily fish) would be prudent as it has been shown to both improve insulin sensitivity and reduce total and low-density lipoprotein-cholesterol.^29^ This would have particularly significant implications for lipid and insulin resistance management in the T2DM group—a population with a twofold risk of cardiovascular disease.^30^

Weight management should be the primary clinical nutritional strategy in T2DM,^4^ and while a range of macronutrient intakes are associated with weight loss in intervention studies, fat remains one of the most important macronutrients in the context of overall energy balance. Data from controlled, ad-lib studies have found evidence of a progressive rise in total EI and body weight on higher per-cent fat diets and the opposite on lower per-cent fat diets (with weight reduction in the order of 0.2–0.3 kg for every 1% reduction in energy from total dietary fat^31, 32, 33^). Behavioural data also suggests that choosing a low-calorie, low-fat diet is a primary dietary strategy among successful long-term weight loss maintainers.^34^ In addition, the beneficial effects of improving fat quality (substituting MUFA for saturated-fat) are not necessarily seen in individuals with a total-fat intake of >37%.^29^ Meats/meat products, butters/full-fat spreads (consumed by over 73% of subjects), eggs and cheeses were among the main contributors to fat and EI in the T2DM cohort. Dietary counseling, which encourages reduced consumption of these foods, substitution of oily fish for meats, and MUFA-rich spreads for butter, with emphasis on appropriate portion sizes for energy balance, would help to reduce the overall energy, total-fat, saturated-fat, sodium and vitamin D intake, and proportionately increase unsaturated-fat intake.

In both the case and control groups, fruit and vegetable intake fell short of the 400 g per day WHO recommendation^7^ and fibre intake was lower than recommended, with significantly lower intake in the T2DM group. Although a high insoluble fibre content in individual foods does not necessarily impact on glycaemic response, broader dietary approaches with a high fibre content (such as the Mediterranean diet) have beneficial effects on glycaemia and weight in T2DM. A high fibre intake is also associated with a decreased mortality risk in T2DM,^35^ and is inversely related to cardiovascular disease and colon cancer.^36^ Increased consumption of plant foods, such as fruit, vegetables and wholegrain cereals (nutrient dense sources of dietary fibre^37^), in T2DM should be encouraged to optimise fibre intake.

Encouraging increased cereal intake in T2DM, however, has the potential to increase the GL of the diet and to elevate plasma trigaclycerol.^23^ Although the GI ranks foods according to their glycaemic effect, the GL represents the overall glycaemic effect of the diet, taking both the GI and amount of carbohydrate consumed into account. Step-wise increases in GL produce proportional increases in blood glucose and insulin,^38, 39^ and reducing dietary GI and GL provides a modest benefit in the clinical management of T2DM^38, 40^ (0.5% reduction in glycosylated haemoglobin^41^). Dietary GL can be reduced in two ways: choosing lower GI carbohydrate foods or reducing total carbohydrate in the diet.^42^ The most significant contributor to GL in the T2DM cohort was wholemeal/brown bread (Figure 1). We have shown previously that the type of wholemeal/brown breads consumed by adults with T2DM in Ireland promote a high glycaemic response.^43^ Advising patients to substitute minimally refined, low-GI foods such as wholegrain pasta and rice, vegetables, fruit and legumes (that currently contribute minimally to GL) for wholemeal/brown breads is likely to increase the fibre content and overall quality of the diet without significantly impacting on GL. This advice also promotes an important component of the Mediterranean diet, proponents of which note that the benefit of whole-grain cereals are in part due to the higher cereal fibre intake, and also to greater ingestion of carbohydrates in a low glycaemic form.^44^ Wholemeal/brown bread and potatoes were also significant contributors to EI in the T2M cohort, and therefore reduced portion size to reflect energy requirements and/or substitution of these foods for more energy dilute foods such as fruit and vegetables would promote energy balance.^45^

In contrast to fat, the T2DM group consumed significantly less total and non-milk sugars than the ND controls. Historically, ‘sugar' intake was restricted in T2DM.^23^ The term ‘sugar' encompasses both naturally occurring sugars that are an intrinsic component of nutrient-dense foods (such as fructose in fruit) and free sugars (sugars naturally present in honey/syrups/fruit juices and glucose/sucrose/ fructose added to foods).^46^ In T2DM, numerous studies have shown no deleterious effects on metabolic control with the addition of sucrose to isocaloric diets.^4, 47^ Consequently, sucrose and other sugars need to be considered primarily from the perspective of energy consumed and substituted for other sources of carbohydrate.^23^ Other authors have noted that for many patients, the public at large, and within the medical community, the notion persists that persons with T2DM should avoid the ingestion of sugars,^23^ leading the International Diabetes Federation to dedicate an event at World Diabetes Day, 2012 to ‘Debunking the Sugar Myth'.^48^ T2DM subjects in the current study consumed significantly less cakes/buns, chocolate confectionary and fruit juices than the ND controls. From this cross-sectional study, we cannot comment on whether food choices in the T2DM subjects were historically different or changed as a result of T2DM diagnosis. A reciprocal relationship however has been noted, between the percentage of energy from dietary fat and that from dietary sugars (a ‘sugar–fat see-saw')^9, 49^ and dietary goals advising a simultaneous reduction in fat and sugar may not be achievable.^50^ Advising patients with T2DM to increase consumption of fibre and micronutrient-dense foods such as fruit and low-fat dairy may increase overall carbohydrate intake, but this may lead to a reciprocal reduction in total and saturated fat intake.

Several of the food choices reported by T2DM subjects appear to promote a higher quality diet than that of ND subjects, for example, more non-white breads and oils. That these choices did not translate to an overall ‘healthier' higher fibre, lower saturated-fat diet, may again relate to broader issues with the options available in the typical western-diet food supply chain.^28^ The food industry produces, and frequently markets, relatively refined grain cereal products and saturated-fat-rich dairy spreads/margarines for their ‘healthfulness'. Wider public health policy change supporting a higher quality food supply and more informative labelling would support T2DM patients' and consumers in general, to make improved food choices.

Previous studies have shown ‘poor compliance' with dietary recommendations in T2DM.^8, 51^ A study of 540 adults with T2DM in Italy^8^ found results similar to our own—a high total and saturated-fat intake and a low fibre intake. This study did not identify the food groups contributing to nutrient intake and therefore it is difficult to translate the findings to food-based recommendations for diabetes educators. On the basis of data from the European Prospective Investigation into Cancer and Multiethnic Cohort studies,^52^ only minor differences in dietary behaviours (primarily related to lower sugar intake) were noted between adults with and without T2DM. The authors called for greater emphasis on education to improve current behaviours but did not specify which dietary messages should be targeted.

All T2DM subjects in the study had diabetes dietary education at diagnosis, with 63% reporting that it was longer than 12 months since they last received dietary advice from an HCP. The type and frequency of diabetes dietary education reported is representative of what is typically described as ‘usual care' in the literature;^53, 54^ access to some diabetes education on diagnosis with *ad hoc* reinforcement of dietary advice at 6- to 12-month intervals. Individuals with T2DM are also exposed to diabetes-related dietary messages from other sources, such as from peers and wider media messages, which may impact on food choices. Interestingly, two decades ago, T2DM subjects in the UK Prospective Diabetes Study were found to have similar intakes to those in the current study.^51^ Despite substantial advances in the medical management of T2DM in the intervening period, this cross-sectional analysis suggests that nutrient intake in T2DM managed in ‘usual care' has not altered substantively. Structured, T2DM education programmes have the potential to improve weight,^53, 54^ glycaemic control,^53^ fruit/vegetable consumption^55^ and reduce the need for diabetes medication.^56^ In addition, follow-up on an annual basis may provide longer term benefits.^56^ The results evident in the current study suggest that, in order to optimise outcomes, more intensive approaches to dietary education are needed throughout the lifespan of T2DM, in addition to medication intensification.^57^

Misreporting of dietary intake is a well-recognised phenomenon in all self-reported dietary assessments, is associated with increasing BMI and has been documented previously in T2DM.^58^ Although the overall degree of energy under-reporting does not appear to have differed significantly between the T2DM and ND cohorts or effected the overall results, it is possible that foods perceived as less ‘socially acceptable' in T2DM may have been under-reported to a greater degree than in the ND subjects. We cannot exclude the possibility that the higher fat and lower carbohydrate intake found may reflect selective under-reporting of sucrose containing foods that may be considered ‘socially undesirable' in T2DM in contrast to fat, which has little effect on glycaemic control. Although every effort was made to minimise the impact of the dietary assessment on habitual intake, weighed intake methods are burdensome and may influence individuals to alter their ‘typical diet'.^59^ Also given the mean BMI >30 kg m^−2^ in both groups, this may have represented typical intake concurrent with a weight loss attempt. The categorisation of foods into food groups with similar characteristics is necessary for this type of analysis and while foods were classified into groups that were relevant to the current study, it must be noted that different categorisation may have affected the outcome of the analysis. Selection bias is an ever-present possibility in all population-based research; however, the subjects included within the present analysis are likely to be representative of the wider population, in terms of age, BMI and glycaemic control, given their similarity to other Irish T2DM populations described in the literature.^60^

## Conclusion

In summary, this study provides a detailed picture of how self-reported food and nutrient intake differs in individuals with T2DM attending a hospital-based diabetes service compared with similar adults without diabetes. Individuals with T2DM consumed a high saturated-fat diet, with a higher total-fat, MUFA, PUFA and protein content and a lower GL, carbohydrate, fibre and sugar content than ND individuals. These findings offer potential insights into foods that require particular focus during dietary education when attempting to improve the overall quality of the diet in T2DM. Dietary education needs to emphasise and regularly reinforce the importance of higher fibre, fruit, vegetable and wholegrain intake and the substitution of monounsaturated for saturated-fat sources, in energy balanced conditions, throughout the lifespan of T2DM.

## Figures and Tables

**Table 1:** Demographic comparison of individuals with type 2 diabetes and with no diabetes

	*Type 2 diabetes*			*No diabetes*			P*-value*
	n	*Median*	*IQR*	n	*Median*	*IQR*	
Age (years)	124	57.7	54.0–61.6	124	57.5	52.0–62.0	0.57
BMI (kg m^−2^)	124	31.9	28.7–35.1	124	30.4	28.5–33.3	0.07
Waist circumference (cm)	115	108.0	100.0–118.0	124	102.0	96.0–110.0	<0.001
Fasting glucose (mmol l^−1^)	121	7.8	6.8–9.5	123	5.6	5.1–6.0	<0.001
Total cholesterol (mmol l^−1^)	123	4.1	3.6–4.6	123	5.2	4.5–5.9	<0.001
HDL-cholesterol (mmol l^−1^)	116	1.1	1.0–1.3	122	1.4	1.2–1.7	<0.001
LDL-cholesterol (mmol l^−1^)	112	2.1	1.6–2.7	121	3.1	2.4–3.6	<0.001
Triglycerides (mmol l^−1^)	123	1.7	1.2–2.5	124	1.3	1.0–2.0	0.002
Systolic BP (mm Hg)	124	133.5	120.0–145.8	119	134.0	123.5–145.5	0.38
Diastolic BP (mm Hg)	124	80.0	70.0–85.0	119	82.5	76.5–90.0	0.002
T2DM diagnosis (years)	124	6.7	4.0–10.5	—	—	—	
HOMAIR-2	121	1.1	1.0–1.4	—	—	—	
HbA1c (mmol mol^−1^)/(%)	122	55/7.2	50–68/6.7–8.4	—	—	—	
	n	*%*		n	*%*		χ*2* P*-value*
*Sex*							1.000
Male	79	63.7		79	63.7		
Female	45	36.3		45	36.3		
							
*Social class*							0.67
Professional, managerial+technical	52	45.6		54	43.9		
Non-manual skilled	24	21.1		28	22.8		
Manual skilled	20	17.5		27	22.0		
Semiskilled+unskilled	18	15.8		14	11.4		
							
*Smoking status*							0.26
Current	21	17.1		16	13.1		
Ex	56	45.5		48	39.3		
Never	46	37.4		58	47.5		
Alcohol consumer	96	77.4		106	86.2		0.08
							
*Nutritional supplements*^a^							
Regular use	21	16.9		39	32.0		0.006
							
*T2DM treatment*							
Diet alone	14	11.3		—	—		
Diet+OHA	86	69.4		—	—		
Diet+OHA+insulin	24	19.4		—	—		

Abbreviations: BMI, body mass index; BP, blood pressure; HbA1c, glycosylated haemoglobin; HDL, high-density lipoprotein; HOMAIR-2, homeostasis model assessment; IQR, interquartile range; LDL, low-density lipoprotein; OHA, oral hypoglycaemic agents; T2DM, type 2 diabetes.

Differences between groups for continuous variables were assessed using the Wilcoxon signed-rank test, except the underlined variables, which were assessed using the paired *t*-test. Categorical variables were assessed using the *χ*^2^-test. *P*<0.05 was considered to be statistically significant.

a Nutritional supplements refers concentrated oral preparations of vitamins, minerals, oils and/or botanical extracts.

**Table 2:** Nutrient intake in individuals with type 2 diabetes and with no diabetes

	*Recommendation*	Type 2 diabetes			No diabetes			P*-value*^a^	P*-value*^b^
		*Median*	*IQR*	*MR %*	*Median*	*IQR*	*MR %*		
*Macronutrients*									
Energy (kcal)		1864.6	1600.6–2230.3	–	1928.0	1542.0–2364.6	–	0.99	
% Energy from protein	10–20^c^	18.6	16.2–20.4	75.4	17.5	15.1–19.7	80.6	0.026	0.20
% Energy from fat	<35^c^	38.8	35.4–44.2	25.4	35.0	30.1–38.0	54.0	<0.001	<0.001
% Energy from saturated fat	<10^c^	13.5	12.0–16.6	5.7	13.6	11.6–16.4	9.6	0.66	0.18
% Energy from PUFA	<10^c^	6.7	5.3–8.1	84.4	5.9	4.7–7.1	96.7	<0.001	0.001
% Energy from MUFA	10–20^c^	13.3	11.3–15.3	89.3	12.2	10.7–13.5	86.3	0.004	0.30
% Energy from carbohydrate	45–60^c^	39.8	37.8–43.5	20.5	43.4	39.3–47.2	44.4	<0.001	<0.001
% Energy from starch		26.2	22.6–30.9	–	25.5	22.1–28.6	–	0.07	–
% Energy from total sugars		12.0	8.7–15.1	–	16.5	14.1–20.8	–	<0.001	–
% Energy from non-milk sugars		10.4	6.7–13.9	–	15.0	11.4–18.4	–	<0.001	<0.001
% Energy from alcohol		0.0	0.0–0.3	–	1.7	0.0–9.0	–	<0.001	–
Dietary fibre (g)	24^d^	14.4	10.8–17.8	12.1	18.9	14.8–24.4	26.6	<0.001	0.002
Glycaemic load		120.8	93.4–144.0	–	129.2	99.7–167.8	–	0.021	–
Glycaemic index		59.9	56.8–63.0	–	60.1	56.9–63.9	–	0.48	–
									
*Micronutrients*									
Sodium (mg)	<2300^e^	2719.8	2243.3–3440.6	27.9	2528.3	1901.9–2969.6	42.7	<0.001	0.01
Calcium (mg)	800^e^	773.9	635.4–961.3	47.5	860.1	702.1–1147.5	59.7	0.008	0.04
Magnesium (mg)		293.4	235.0–346.1	–	280.5	235.8–352.6	–	0.83	–
Iron (mg)	♂ 10/♀ 9–14^e^	12.2	10.3–15.5	66.4	12.1	9.4–15.3	59.7	0.23	0.17
Vitamin D (ug)	10^e^	3.0	1.9–4.7	2.5	3.2	1.9–5.6	12.9	0.07	0.002
Vitamin B12 (μg)	1.4^e^	4.9	3.6–6.8	99.2	4.9	3.7–7.0	98.4	0.16	0.51
Folate (μg)	300^e^	317.2	233.2–437.8	53.3	306.8	246.8–435.4	54.0	0.78	0.50
Vitamin C (mg)	60^e^	74.9	39.6–103.7	44.3	75.9	50.7–114.4	33.9	0.13	0.06

Abbreviations: IQR, interquartile range; %MR, % meeting recommendations; MUFA, monounsaturated fatty acids; PUFA, polyunsaturated fatty acids.

*P*<0.05 was considered to be statistically significant.

a *P*-value differences between groups for median intake were assessed using the Wilcoxon signed-rank test, except the underlined variables that were assessed using the paired *t*-test for mean differences.

b *P*-value differences in the % meeting recommendations between the two groups were assessed using the *χ*^2^-test.

c Recommendations from The Diabetes and Nutrition Study Group of the European Association for the Study of Diabetes (2004).

d Recommendations from Diabetes UK (2011).

e Recommendations from Irish Recommended Dietary Allowances (1999).

**Table 3:** Food group intake in individuals with type 2 diabetes and with no diabetes

*Food group (g per day)*	*T2DM*					*ND*					P*-value*
	*Total population (*n*=124)*			*Consumers only*		*Total population (*n*=124)*			*Consumers only*		
	*Median*	*IQR*	*% Cons*	*Median*	*IQR*	*Median*	*IQR*	*% Cons*	*Median*	*IQR*	
White bread and rolls	21.8	0.0–353.8	69.7	39.0	3.5–353.8	48.3	0.0–286.5	79.8	62.5	5.3–286.5	0.002
Wholemeal and brown bread and rolls	59.3	0.0–329.8	82.0	73.5	5.0–329.8	40.5	0.0–237.5	71.0	61.8	3.8–237.5	0.03
Wholegrain breads	0.0	0.0–207.0	33.6	54.5	8.0–207.0	0.0	0.0–202.0	18.6	43.0	5.0–202.0	0.006
Pasta/rice/grains	0.0	0.0–293.8	43.4	57.5	12.5–293.8	0.0	0.0–187.0	48.4	44.3	6.3–187.0	0.88
Refined breakfast cereals	0.0	0.0–66.0	18.9	15.8	5.3–66.0	0.0	0.0–118.8	29.8	20.0	5.0–118.8	0.045
Wholegrain breakfast cereals	21.3	0.0–369.5	67.2	37.5	9.0–369.5	36.6	0.0–737.5	64.5	85.0	8.3–737.5	0.001
Biscuits	3.4	0.0–54.3	55.7	13.0	2.5–54.3	4.4	0.0–137.3	54.8	18.0	1.8–137.3	0.39
Cakes/pastries/buns	0.0	0.0–164.3	41.0	38.5	5.8–164.3	20.0	0.0–232.5	62.9	33.8	1.3–232.5	0.001
Cream/ice cream/desserts	0.0	0.0–168.8	50.0	42.5	7.5–168.8	0.0	0.0–206.8	49.2	35.3	1.0–206.8	0.87
Full-fat milk and yogurts	0.0	0.0–677.5	37.7	70.6	8.8–677.5	65.6	0.0–805.8	77.4	92.5	1.3–805.8	<0.001
Low-fat milk and yoghurts	103.8	0.0–736.3	70.5	165.0	17.5–736.3	45.4	0.0–875.5	58.9	150.0	3.3–875.5	0.14
Cheeses	10.0	0.0–86.3	62.3	15.0	2.5–86.3	7.4	0.0–100.0	63.7	15.0	3.0–100.0	0.37
Sugars, syrups, preserves and sweeteners	0.0	0.0–56.3	39.3	10.4	0.8–56.3	6.9	0.0–96.3	72.6	12.0	0.5–96.3	<0.001
Chocolate confectionary	0.0	0.0–70.5	23.0	13.5	1.3–70.5	0.0	0.0–67.5	36.3	13.0	2.3–67.5	0.049
Non-chocolate confectionary	0.0	0.0–22.8	4.1	15.0	7.0–22.8	0.0	0.0–62.8	16.9	12.5	0.8–62.8	0.001
Eggs and egg dishes	15.3	0.0–120.0	65.6	30.0	12.5–120.0	15.0	0.0–149.8	59.7	30.0	5.3–149.8	0.022
Butter/full-fat spreads	11.6	0.0–80.0	73.0	19.3	1.8–80.0	9.1	0.0–75.0	75.0	14.0	1.3–75.0	0.06
Lower fat spreads	0.0	0.0–72.5	34.4	24.6	2.5–72.5	0.0	0.0–102.0	33.9	11.3	0.5–102.0	0.21
Oils	0.0	0.0–27.8	26.2	2.8	0.8–27.8	0.0	0.0–11.3	8.1	3.0	0.3–11.3	<0.001
Potatoes including processed/homemade potato	89.4	0.0–409.0	91.0	105.0	15.0–409.0	78.3	0.0–353.5	85.5	95.0	17.0–353.5	0.19
Chipped, fried and roasted potato	37.5	0.0–188.8	64.8	50.0	10.0–188.8	31.1	0.0–214.8	66.9	50.5	10.5–214.8	0.90
Peas/beans/lentils	20.1	0.0–150.0	66.4	39.8	10.0–150.0	14.6	0.0–156.5	63.7	30.0	6.0–156.5	0.06
Other vegetables	108.0	0.0–370.0	97.5	110.0	15.0–370.0	88.4	0.0–395.0	98.4	90.5	0.5–395.0	0.038
Fruit	105.0	0.0–864.0	79.5	138.5	13.3–864.0	81.4	0.0–699.0	81.5	94.8	5.5–699.0	0.58
Fruit juices	0.0	0.0–323.3	17.2	62.5	10.0–323.3	0.0	0.0–395.0	39.5	100.0	2.5–395.0	<0.001
White fish/shellfish	0.0	0.0–144.3	44.3	53.6	11.3–144.3	0.0	0.0–210.8	43.6	39.8	8.3–210.8	0.84
Oily fish	0.0	0.0–79.8	23.8	39.0	12.5–79.8	0.0	0.0–110.0	32.3	38.4	6.3–110.0	0.039
Poultry	33.8	0.0–201.5	68.0	58.8	9.8–201.5	40.6	0.0–250.5	74.2	63.3	6.3–250.5	0.58
Red meat	42.5	0.0–194.8	74.6	51.3	12.5–194.8	44.4	0.0–418.8	71.0	66.5	3.0–418.8	0.40
Other meat/meat products	47.8	0.0–260.0	94.3	52.5	5.8–260.0	38.6	0.0–212.3	91.1	46.0	3.0–212.3	0.019
Savoury snacks	0.0	0.0–63.0	29.5	10.0	2.5–63.0	0.0	0.0–23.0	17.7	9.5	1.3–23.0	0.049
Herbs/spices/nuts & seeds	0.0	0.0–45.0	17.2	2.5	0.3–45.0	0.0	0.0–39.3	10.5	10.0	0.5–39.3	0.73
Soups & sauces	52.4	0.0–402.5	86.1	60.8	3.0–402.5	34.4	0.0–397.0	78.2	55.0	0.8–397.0	0.038
Savouries	0.0	0.0–330.0	44.3	78.8	15.0–330.0	0.0	0.0–174.0	37.9	87.0	7.5–174.0	0.50
Alcoholic beverages	0.0	0.0–1291.5	35.3	250.0	11.5–1291.5	49.5	0.0–3051.0	58.1	221.9	8.8–3051.0	0.005
Low energy beverages	900.6	0.0–3170.0	100.0	922.5	47.5–3170.0	974.4	0.0–5400.0	100.0	974.4	155.3–5400.0	0.34
High energy beverages	0.0	0.0–562.5	23.8	150.0	12.5–562.5	0.0	0.0–659.3	33.1	134.3	4.5–659.3	0.50

Abbreviations: % Cons, percentage consumers; IQR, interquartile range; ND, no diabetes; T2DM, type 2 diabetes.

Differences in median intake between the T2DM and ND group (total population) were assessed using the Wilcoxon signed-rank test. *P*<0.05 was considered to be statistically significant.

**Table 4:** Food group contribution to nutrient intake in individuals with T2DM and with ND

*Food group*	*% Contribution to energy*		P*-value*	*% Contribution to carbohydrate*		P*-value*	*% Contribution to protein*		P*-value*	*% Contribution to total fat*		P*-value*
	*T2DM*	*ND*		*T2DM*	*ND*		*T2DM*	*ND*		*T2DM*	*ND*	
	*Median (IQR)*	*Median (IQR)*		*Median (IQR)*	*Median (IQR)*		*Median (IQR)*	*Median (IQR)*		*Median (IQR)*	*Median (IQR)*	
White bread and rolls	2.7 (0.0–7.7)	6.0 (2.2–10.9)	0.002	4.8 (0.0–14.5)	11.3 (3.5–18.9)	0.005	2.0 (0.0–5.3)	5.0 (1.5–9.4)	0.001	0.9 (0.0–1.8)	1.5 (0.4–3.6)	0.002
Wholemeal and brown bread and rolls	6.6 (1.8–11.9)	4.1 (0.0–9.5)	0.027	11.3 (3.1–21.2)	6.9 (0.0–16.8)	0.020	5.2 (1.7–10.3)	4.0 (0.0–8.8)	0.11	1.8 (0.4–4.5)	1.0 (0.0–2.3)	0.001
Wholegrain breads	0.0 (0.0–3.2)	0.0 (0.0–0.0)	0.005	0.0 (0.0–5.7)	0.0 (0.0–0.0)	0.003	0.0 (0.0–2.5)	0.0 (0.0–0.0)	0.005	0.0 (0.0–0.9)	0.0 (0.0–0.0)	0.014
Pasta/rice/grains	0.0 (0.0–3.2)	0.0 (0.0–3.0)	0.81	0.0 (0.0–6.0)	0.0 (0.0–5.7)	0.68	0.0 (0.0–1.7)	0.0 (0.0–1.7)	0.99	0.0 (0.0–0.6)	0.0 (0.0–0.7)	0.49
Refined breakfast cereal	0.0 (0.0–0.0)	0.0 (0.0–2.0)	0.06	0.0 (0.0–0.0)	0.0 (0.0–3.5)	0.09	0.0 (0.0–0.0)	0.0 (0.0–1.1)	0.032	0.0 (0.0–0.0)	0.0 (0.0–0.2)	0.06
Wholegrain breakfast cereals	3.9 (0.0–7.5)	3.4 (0.0–7.3)	0.74	6.8 (0.0–13.3)	5.7 (0.0–11.1)	0.17	2.7 (0.0–4.8)	2.4 (0.0–5.3)	0.56	1.2 (0.0–3.5)	1.1 (0.0–3.8)	0.33
Biscuits	0.9 (0.0–3.3)	0.9 (0.0–4.5)	0.50	1.2 (0.0–4.5)	1.3 (0.0–5.6)	0.65	0.2 (0.0–1.0)	0.3 (0.0–1.4)	0.43	0.7 (0.0–3.2)	1.1 (0.0–4.9)	0.13
Cakes/pastries/buns	0.0 (0.0–5.3)	3.1 (0.0–7.9)	0.002	0.0 (0.0–7.8)	3.6 (0.0–10.1)	0.010	0.0 (0.0–1.7)	1.2 (0.0–3.5)	0.003	0.0 (0.0–3.8)	3.1 (0.0–8.0)	<0.001
Cream/icecream/desserts	0.0 (0.0–3.7)	0.0 (0.0–3.5)	0.69	0.0 (0.0–3.8)	0.0 (0.0–3.4)	0.81	0.0 (0.0–1.4)	0.0 (0.0–1.4)	0.74	0.0 (0.0–5.3)	0.0 (0.0–4.8)	0.50
Full-fat milk and yogurts	0.0 (0.0–1.6)	2.5 (0.3–5.3)	<0.001	0.0 (0.0–1.6)	2.4 (0.2–5.3)	<0.001	0.0 (0.0–1.5)	2.7 (0.3–6.5)	<0.001	0.0 (0.0–1.9)	2.7 (0.4–6.3)	0.000
Low-fat milk and yoghurts	2.4 (0.0–5.0)	1.3 (0.0–4.2)	0.10	2.3 (0.0–5.6)	1.2 (0.0–4.6)	0.025	4.2 (0.0–8.6)	2 (0.0–7.4)	0.23	1.4 (0.0–3.5)	0.7 (0.0–3.2)	0.52
Cheeses	1.7 (0.0–3.5)	1.4 (0.0–3.3)	0.72	0.0 (0.0–0.0)	0.0 (0.0–0.0)	0.060	2.3 (0.0–5.2)	2.1 (0.0–5.1)	0.67	3.0 (0.0–6.2)	2.7 (0.0–7.3)	0.31
Sugars, syrups, preserves and sweeteners	0.0 (0.0–0.8)	0.9 (0.0–3.5)	<0.001	0.0 (0.0–2)	2.2 (0.0–8.3)	<0.001	0.0 (0.0–0.0)	0.0 (0.0–0.0)	0.005	0.0 (0.0–0.0)	0.0 (0.0–0.0)	0.039
Chocolate confectionary	0.0 (0.0–0.0)	0.0 (0.0–2.5)	0.031	0.0 (0.0–0.0)	0.0 (0.0–2.4)	0.07	0.0 (0.0–0.0)	0.0 (0.0–0.8)	0.014	0.0 (0.0–0.0)	0.0 (0.0–3.4)	0.007
Non-chocolate confectionary	0.0 (0.0–0.0)	0.0 (0.0–0.0)	<0.001	0.0 (0.0–0.0)	0.0 (0.0–0.0)	0.001	0.0 (0.0–0.0)	0.0 (0.0–0.0)	0.003	0.0 (0.0–0.0)	0.0 (0.0–0.0)	0.017
Eggs and egg dishes	1.6 (0.0–3.4)	1.1 (0.0–2.8)	0.06	0.0 (0.0–0.0)	0.0 (0.0–0.0)	0.38	2.7 (0.0–5.3)	1.9 (0.0–4.9)	0.09	3.1 (0.0–5.9)	2.5 (0.0–5.6)	0.37
Butter/full-fat spreads	3.8 (0.0–9.4)	2.8 (0.1–6.3)	0.041	0.0 (0.0–0.1)	0.0 (0.0–0.1)	0.007	0.1 (0.0–0.2)	0.1 (0.0–0.1)	0.08	9.1 (0.0–22.9)	8.1 (0.4–19.1)	0.31
Lower fat spreads	0.0 (0.0–2.8)	0.0 (0.0–1)	0.15	0.0 (0.0–0.1)	0.0 (0.0–0.0)	0.042	0.0 (0.0–0.0)	0.0 (0.0–0.0)	0.60	0.0 (0.0–7.3)	0.0 (0.0–2.7)	0.28
Oils	0.0 (0.0–0.3)	0.0 (0.0–0.0)	0.001	0.0 (0.0–0.0)	0.0 (0.0–0.0)	1.000	0.0 (0.0–0.0)	0.0 (0.0–0.0)	1.000	0.0 (0.0–0.7)	0.0 (0.0–0.0)	0.001
Potatoes including processed/homemade potato	4.0 (2.4–6.3)	3.6 (1.6–5.6)	0.027	7.5 (4.5–11.9)	6.3 (2.7–9.7)	0.07	1.8 (1.1–2.8)	1.8 (0.9–3.0)	0.59	0.5 (0.1–3.2)	0.2 (0.1–1.7)	0.001
Chipped, fried and roasted potato	2.8 (0.0–6.0)	2.8 (0.0–6.1)	0.85	4.2 (0.0–7.9)	3.7 (0.0–7.5)	0.78	1 (0.0–2.1)	1.2 (0.0–2.4)	0.53	2 (0.0–5.5)	3.1 (0.0–8.2)	0.049
Peas/beans/Lentils	0.6 (0.0–2.2)	0.5 (0.0–1.4)	0.033	0.9 (0.0–3.2)	0.7 (0.0–1.7)	0.011	1.1 (0.0–3.0)	1.0 (0.0–2.2)	0.08	0.2 (0.0–0.4)	0.2 (0.0–0.4)	0.46
Other vegetables	2.0 (1.0–3.7)	1.7 (0.8–3.1)	0.09	2.1 (1.4–3.7)	1.6 (0.7–2.7)	<0.001	1.5 (0.8–2.5)	1.3 (0.8–2.4)	0.49	1.4 (0.5–4.1)	1.6 (0.4–5.0)	0.83
Fruit	3.2 (0.7–5.3)	2.5 (1.0–4.5)	0.28	7.9 (1.7–12.2)	5.5 (2.3–9.9)	0.12	0.9 (0.2–1.6)	0.7 (0.3–1.3)	0.40	0.2 (0.0–0.4)	0.2 (0.1–0.4)	0.63
Fruit juices	0.0 (0.0–0.0)	0.0 (0.0–1.2)	<0.001	0.0 (0.0–0.0)	0.0 (0.0–2.7)	0.001	0.0 (0.0–0.0)	0.0 (0.0–0.3)	<0.001	0.0 (0.0–0.0)	0.0 (0.0–0.1)	0.001
White fish/shellfish	0.0 (0.0–3.4)	0.0 (0.0–3.6)	0.82	0.0 (0.0–0.7)	0.0 (0.0–1.6)	0.78	0.0 (0.0–8.4)	0.0 (0.0–7.5)	0.75	0.0 (0.0–2.6)	0.0 (0.0–4.4)	0.24
Oily fish	0.0 (0.0–0.0)	0.0 (0.0–2.7)	0.007	0.0 (0.0–0.0)	0.0 (0.0–0.0)	0.09	0.0 (0.0–0.0)	0.0 (0.0–7.7)	0.008	0.0 (0.0–0.0)	0.0 (0.0–3.4)	0.011
Poultry	3.7 (0.0–6.7)	3.3 (0.0–6.5)	0.96	0.0 (0.0–0.0)	0.0 (0.0–1.1)	0.001	11.1 (0.0–20.7)	11.0 (0.0–21.6)	0.67	3.3 (0.0–7.0)	2.3 (0.0–8.1)	0.43
Red meat	4.8 (0.0–9.2)	4.2 (0.0–8.1)	0.26	0.0 (0.0–0.0)	0.0 (0.0–0.0)	0.002	11.6 (0.0–21.4)	12.6 (0.0–21.9)	0.99	7.1 (0.0–14.4)	5.2 (0.0–9.6)	0.037
Other meat/meat products	6.6 (2.8–11.1)	4.7 (1.8–8.8)	0.009	0.4 (0.1–1.5)	0.1 (0.0–1.8)	0.21	11.3 (6.7–18.3)	8.9 (4.3–14.7)	0.004	10.3 (3.6–16.7)	7.5 (2.2–13.6)	0.014
Savoury snacks	0.0 (0.0–1.0)	0.0 (0.0–0.0)	0.040	0.0 (0.0–0.5)	0.0 (0.0–0.0)	0.14	0.0 (0.0–0.3)	0.0 (0.0–0.0)	0.09	0.0 (0.0–1.6)	0.0 (0.0–0.0)	0.053
Herbs/spices/nuts and seeds	0.0 (0.0–0.0)	0.0 (0.0–0.0)	0.76	0.0 (0.0–0.0)	0.0 (0.0–0.0)	0.78	0.0 (0.0–0.0)	0.0 (0.0–0.0)	0.91	0.0 (0.0–0.0)	0.0 (0.0–0.0)	0.89
Soups and sauces	2.5 (0.5–4.0)	1.3 (0.2–3.2)	0.012	1.4 (0.3–3.7)	0.9 (0.1–2.1)	0.007	0.8 (0.1–2.0)	0.5 (0.0–1.2)	0.008	2.2 (0.4–6.6)	1.6 (0.0–4.0)	0.032
Savouries	0.0 (0.0–5.6)	0.0 (0.0–4.5)	0.25	0.0 (0.0–4.9)	0.0 (0.0–2.6)	0.20	0.0 (0.0–6.7)	0.0 (0.0–6.9)	0.64	0.0 (0.0–6.0)	0.0 (0.0–5.7)	0.33
Alcoholic beverages	0.0 (0.0–3.5)	1.9 (0.0–9.5)	<0.001	0.0 (0.0–0.1)	0.0 (0.0–1.7)	0.002	0.0 (0.0–0.1)	0.1 (0.0–0.6)	0.007	0.0 (0.0–0.0)	0.0 (0.0–0.0)	1.000
Low energy beverages	0.0 (0.0–0.2)	0.0 (0.0–0.1)	0.002	0.0 (0.0–0.2)	0.0 (0.0–0.1)	0.001	0.6 (0.3–1.1)	0.8 (0.5–1.1)	0.08	0.0 (0.0–0.0)	0.0 (0.0–0.0)	0.47
High energy beverages	0.0 (0.0–0.0)	0.0 (0.0–1.5)	0.08	0.0 (0.0–0.0)	0.0 (0.0–2.2)	0.022	0.0 (0.0–0.0)	0.0 (0.0–0.0)	0.46	0.0 (0.0–0.0)	0.0 (0.0–0.0)	0.93

Abbreviations: IQR, interquartile range; ND, no diabetes; T2DM, type 2 diabetes.

Differences in median % contribution to nutrient intake between the T2DM and ND group were assessed using the Wilcoxon signed-rank test. *P*<0.05 was considered to be statistically significant.
