# Supplementary material for: High saturated-fat and low-fibre intake: a comparative analysis of nutrient intake in individuals with and without type 2 diabetes
Source: Nutr Diabetes. 2014 Feb 3;4(2):e104–. doi: 10.1038/nutd.2014.2 (PMC3940826; doi:10.1038/nutd.2014.2)
Supplement: Supplementary Appendix 1 [file nutd20142x1.doc]

**Appendix 1: The thirty-eight food groups considered for identifying food group contribution to nutrient intake**

| **Food Group** | **Foods within each food group** |
| --- | --- |
| White bread & rolls | All breads made from white flour including French stick, white pitta, cream crackers, crispbread, naan bread, breadcrumbs, ciabatta and chapatti |
| Wholemeal & brown bread & rolls | All breads made with brown/wholemeal flour including soda bread, wholemeal breads and crackers |
| Wholegrain breads | All breads and rolls made from wholegrain flours including granary bread and rye bread |
| Pasta/Rice/Grains | All pasta, rice, noodles and grains such as couscous, quinoa, barley, buckwheat, bulgar wheat, cassava, tapioca and flours such as wheat/soya/rice/potato, etc |
| Refined breakfast cereals | Cornflakes, Rice Krispies, Crunchy Nut Cornflakes, Special K, Frosties, Cocopops, Ricicles, Smacks, Honey Smacks, Weetos, Start, Sugar Puffs, Cereal Bars |
| Wholegrain breakfast cereals | Branflakes, All-Bran, Wheat and Oat bran, Porridge, Oatflakes, Weetabix, Weetaflakes, Sultana Bran, Fruit’n Fibre, Cheerios, Ready Brek, Mueslis, Puffed Wheat, Grapenuts, Shredded Wheat |
| Biscuits | All biscuits (Digestives, Hobnobs, Jaffa cakes, etc) |
| Cakes/ Pastries/ Buns | Buns (Hot Cross buns, Currant buns), Cakes (Carrot cake, Sponge cake etc.) and pastries (Danish pastries, croissants etc.) |
| Cream/Ice-cream/Desserts | All creams (fresh/whipping/UHT); all ice creams and all deserts and puddings (apple pies, cheesecakes, custard, milkshakes, trifle, banoffee pie, crème caramel, mousse, etc) |
| Full fat milk & yogurts | All full fat cows/goats/sheep/soya/rice milks and yoghurts (including fresh, dried, UHT, evaporated and condensed milks, Lassi and Tzatziki) |
| Low fat milk & yoghurts | All low fat and skimmed cows/goats/sheeps/soya milks and yoghurts (including fresh, dried, UHT, evaporated and condensed milks, Lassi and Tzatziki) |
| Cheeses | All cheeses  (Cheddar, Mozzerella, Brie, Camembert, Cream Cheese, Goats Cheese, Cheese spreads and processed cheeses) |
| Sugars, syrups, preserves & sweeteners | All sugars (white/brown); jams and marmalades, honey, molasses, treacle, salt and vinegar |
| Chocolate confectionary | All chocolate, chocolate bars and chocolate spreads |
| Non-chocolate confectionary | All non-chocolate sweets including boiled sweets, non-chocolate cereal bars, fudge, jellies, licorice, marshmallows |
| Eggs and egg dishes | All eggs (chickens/duck/quail) and all egg dishes (omelette, quiche, etc) |
| Butter/full fat spreads and margarines | All butter and spreads that contain > 40% fat (e.g. Dairygold Original, Flora) |
| Lower fat spreads and margarines | All spreads that contain 40% fat or less (e.g. Low Low, Flora light) |
| Oils | All oils (vegetable oils, olive oil, coconut oil, etc) |
| Potatoes including processed and homemade potato products | All boiled/baked/mashed potatoes including sweet potato, yam and plantain |
| Chipped, fried & roasted potato | All fried/roasted/chipped potatoes including potato wedges, potato cakes, potato fritters, potato waffles, hash browns, potato croquettes |
| Peas / Beans / Lentils | All high protein vegetables including beans and bean products (tofu), lentils and pulses (peas, sweetcorn) |
| Other vegetables | All root vegetables (carrots, parsnips) and green leafy vegetables. |
| Fruit | All kinds of fruit (fresh/canned/dried) |
| Fruit juices | All kinds of fruit juice, freshly squeezed or bottled |
| White fish/Shellfish | All kinds of white fish and shell fish (cod, haddock, plaice, whiting, canned tuna, crab, mussels, etc). Also includes products like fish pate and fish paste. |
| Oily fish | All kinds of oily fish (salmon, trout, mackerel, eel, kipper, sardines, fresh tuna, swordfish, anchovies, herring etc) |
| Poultry | Includes all poultry and game birds (chicken, turkey, pheasant, pigeon, duck, goose) |
| Red meat | Includes all red meats and game mammals (beef, lamb, venison, rabbit, pork, mutton) |
| Other meat/meat products | Includes all processed meats (bacon, ham, sausages, pudding); offal (liver and liver pate); meat products (nuggets, kebabs, burgers) and meat pies |
| Savoury snacks | All savoury snacks such as potato crisps, popcorn, nuts, seeds, pretzels |
| Herbs/spices/nuts & seeds | All herbs (fresh and dried), spices, nuts and seeds |
| Soups & sauces | Includes all soups, broths and consommés and sauces (white sauces, pasta sauces, gravy, chutneys, relish, salad dressings etc) |
| Savouries | All savoury dishes such as Indian and Chinese dishes, Savoury Pancakes, Stews, Casseroles, Shepard’s Pie, etc.) |
| Alcoholic beverages | Includes all alcoholic beverages except non-alcoholic lager |
| Low energy beverages | Includes water, tea, coffee, sugar free cordials and diet fizzy drinks |
| High energy beverages | Includes non-diet fizzy drinks, fruit squashes/cordials, hot chocolate, malted drinks made on milk, etc |
| Nutritional supplements | Includes multivitamin/mineral supplements, single vitamins, single minerals, fish oils and other oils taken in addition to food |
